# Experimental study on the effects of bismuth subgallate on the inflammatory process and angiogenesis of the oral mucosa^☆^^☆☆^

**DOI:** 10.1016/j.bjorl.2014.12.009

**Published:** 2015-10-27

**Authors:** Eduardo Vieira Couto, Carlos Roberto Ballin, Claudia Paraguaçu Pupo Sampaio, Carlos Augusto Seije Maeda, Carlos Henrique Ballin, Camila Soares Dassi, Lilian Yukari Miura

**Affiliations:** a Department of Otorhinolaryngology, Hospital Universitário Evangélico de Curitiba (HUEC), Curitiba, PR, Brazil; b Department of Surgery, Universidade Federal do Paraná (UFPR), Curitiba, PR, Brazil; c Department of Surgery, Pontifícia Universidade Católica do Paraná (PUCPR), Curitiba, PR, Brazil; d Functional Rhinology, Irmandade Santa Casa de Misericórdia de Curitiba, Curitiba, PR, Brazil; e Department of Otorhinolaryngology, Pontifícia Universidade Católica do Paraná (PUCPR), Curitiba, PR, Brazil

**Keywords:** Wound healing, Tonsillectomy, Angiogenesis-inducing agents, Cicatrização, Tonsilectomia, Indutores da angiogênese

## Abstract

**Introduction:**

Bismuth subgallate is a salt derived from heavy metal. The aim of this study was to evaluate the effect of this salt on some phases of healing.

**Objectives:**

To assess the effect of subgallate on mucosa and to evaluate the association between the use of bismuth subgallate and neogenesis of vessels in oral mucosal wounds.

**Methods:**

This was a prospective and experimental study. This study used sixty rats, which were divided into control and experimental groups. The animals were submitted to a surgical procedure, which caused oral mucosal injury. A saline solution was applied on the wound of the control group, and in the experimental group, a solution of bismuth subgallate was administrated.

**Results:**

The experimental group showed greater inflammatory reaction with increasing monomorphic proliferation. There was increased vessel proliferation in the control group.

**Conclusion:**

Bismuth subgallate had a negative influence on the healing process, delaying the rate of new vessel formation and optimal wound healing.

## Resumo

**Introdução:**

O subgalato de bismuto é um sal derivado de metal pesado. A ideia desta pesquisa é avaliar sua interferência em alguma das fases da cicatrização.

**Objetivo:**

Delinear a ação do subgalato em mucosas. Avaliar a relação entre a utilização do subgalato de bismuto e a neoformação de vasos nas feridas em mucosa oral, para evidenciar o possível benefício resultante do seu uso.

**Método:**

Estudo experimental, prospectivo. Utilizou-se sessenta ratos, que foram divididos igualmente em grupo controle e experimento. Foram submetidos a um procedimento cirúrgico onde foi feito uma lesão na mucosa oral dos animais, após, uma solução de soro fisiológico foi aplicada sobre a lesão do grupo controle e sobre a ferida do grupo experimento foi aplicada uma solução de subgalato de bismuto.

**Resultados:**

o grupo experimento apresentou maior reação inflamatória com crescente proliferação monomórfica. Vasos: houve maior proliferação no grupo controle.

**Conclusões:**

concluiu-se que o subgalato de bismuto teve uma ação negativa no processo de cicatrização, atrasando a velocidade de formação dos neovasos e a cicatrização ideal da ferida operatória.

## Introduction

Bismuth subgallate or bismuth oxygallate is a yellowish substance, which presents as an odorless powder that suffers discoloration in the presence of sunlight.^1^ It is a heavy metal salt that is poorly absorbed, and has strong astringent power.^2^

Dermatol, another name for the salt, has been increasingly used by professionals from the otorhinolaryngology and dentistry areas. Due to its hemostatic and astringent properties, the substance may be used in many ways; among them, as topical treatment for open wounds, treatment of gastroduodenal ulcers, diarrhea, odor control in colostomies, in dental surgeries, epistaxis management and, empirically, in adenotonsillectomies.

The literature is not in unified opinion about the benefits of subgallate in hemostasis or the healing effect and there are discrepancies among studies. It does not address whether there are beneficial effects on healing, and there are few studies with comparison or control groups. However, Arroyo Júnior et al.^2^ found that bismuth subgallate has an effect on factor 12 activation, which accelerates the intrinsic pathway in the coagulation cascade, contributing to the hemostatic characteristic of this compound. The authors also discussed the possibility that bismuth subgallate might inhibit fibrinolysis and, thereby, increase fibrosis.

According to Hernández-Paz et al.^3^ (from the otolaryngology service of the outpatient surgery center ISSSTE, in Mexico City, Mexico), some methods are currently being investigated to reduce or prevent intraoperative or postoperative bleeding during adenotonsillectomy. For this reason, several means have been compared, such as a laser, electrodissection, cold ablation, harmonic scalpel, or radio frequency. However, none of these techniques showed 100% efficacy. In this search for an ideal method, two substances derived from bismuth (subgallate, and bismuth subsalicylate) have been reported, which exhibit hemostatic effects that activate factor XII *via* the intrinsic coagulation pathway. When applied topically to the wound, immediately after tissue removal, bleeding is significantly decreased.

Padilla et al.^4^ (from the department of otorhinolaryngology of Centro Médico Nacional La Raza, IMSS, Mexico City, Mexico) described bismuth subgallate as a heavy metal that accelerates clot formation and improves hemostasis. They also reported that some authors have suggested only adrenaline only as the active ingredient that helps hemostasis, due to its vasoconstrictor effect. According to Padilla et al., there have been few studies using bismuth subgallate alone (without adrenaline) to demonstrate its hemostatic effect and, considering that, they proposed a clinical trial comparing the hemostatic effect of subgallate in adenotonsillectomies, compared to a control group that used only dry gauze. In the present study, bismuth subgallate showed no activity as a hemostatic agent to reduce intraoperative bleeding.

The goal of this study was to assess whether bismuth subgallate interferes in any of the healing stages. As it is an inexpensive easy-to-handle powder, its use could be beneficial from the perspective of healing, if case studies document that benefit.

Many otorhinolaryngology services do not use bismuth subgallate, reporting that the immediate postoperative results of its use show no benefits, whereas others report that it helps to control bleeding. The studies, in general, focus on its use in relation to immediate postoperative bleeding control.

Wound healing occurs in several simultaneous processes, and it is not possible to distinguish the beginning or the end of each; however, for didactic purposes, it can be divided into stages: coagulation, inflammation, proliferation, contraction, and remodeling.^1^ The phase of proliferation is subdivided into re-epithelialization, fibroplasia, and angiogenesis.

As angiogenesis is an important healing phase, if bismuth subgallate induces increased angiogenic activity, early and increased neovascularization is expected, culminating in an optimization of the healing process.

Angiogenesis occurs from preexisting vessels, in serum, and in the extracellular matrix. These new vessels assist in granulation tissue formation, as well as by supplying nutrients and oxygen to tissues, thus they are crucial for the healing process, corresponding to 60% of tissue repair.^5^, ^6^

The formation of new vessels is essential for tissue regeneration and, therefore, local factors, chemical mediators, extracellular matrix, and metabolic gradients show concomitant development.^7^

Among the objectives of this study, the general one was outlining subgallate action on mucosal tissue and as a specific objective, the assessment of the association between the use of bismuth subgallate and the formation of new vessels in oral mucosa wounds, thus showing the possible resulting benefits of its use, so that new protocols can finally be utilized in otorhinolaryngology services involving the use of this salt, mainly in oral cavity surgeries.

## Methods

### Surgical procedure

The experiments were carried out from September 24 to October 1, 2012. A total of 60 young adult male Wistar rats (*Rattus norvegiccus albinus*), aged 110 days and mean weight between 250 g and 300 g, were assessed. The sample size was estimated according to studies in the literature and the project followed the guidelines of Law 11,797 of October 8, 2008, regulated by Decree No. 6899 of 15 July 2009 and the recommendations of the Brazilian College of Animal Experimentation. The study was performed after approval by the ethics committee on animal studies under No. 666.

**Figure 1:**
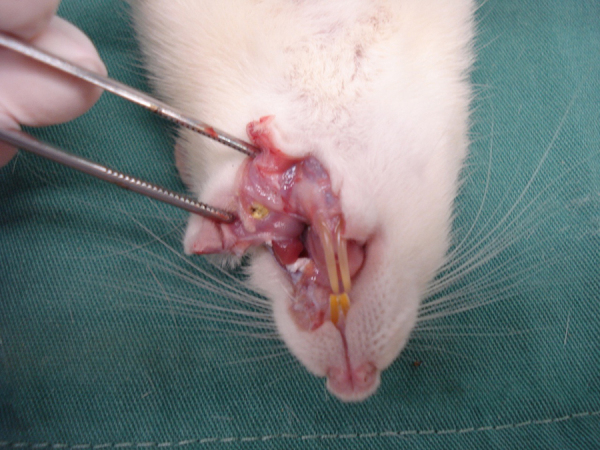
Surgical wound induction.

The animals were anesthetized with 0.1 mL/100 g body weight with a mixture of 1 mL of ketamine (50 mg) and 1 mL xylazine 2% (20 mg), IM, in the posterior portion of the thigh. Subsequently, they were placed in the prone position and had the lower and upper limbs attached to a wooden support (Fig. 1). With the aid of a suitable mouth opener, a wound of approximately 0.7 cm in diameter was made on the left side of the oral mucosa, by using a biopsy forceps.

At this point, it was decided to create a single wound to reduce the aggression to the animals and to facilitate their recovery; the left side was chosen to standardize the study. It was decided to use the biopsy forceps instead of a punch biopsy device due to the chance that the second tool would completely pierce the rat's oral cavity wall. After the surgery, the animals received dipyrone (at a dose of 10 mg/kg) IM for analgesia, according to what was proposed to and accepted by CEUA.

**Figure 2:**
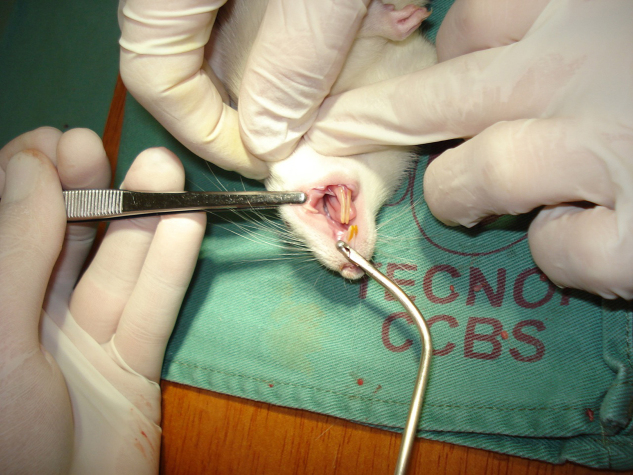
Wound removal on D7.

The animals were randomly marked and divided into three groups of 10 animals, which received 0.5 mg of topic bismuth subgallate after wound with biopsy forceps. Each group was reoperated for wound removal and evaluation after 24 h (GII), three days (GIII), and seven days (GIV) after the lesion (Fig. 2). The remaining 30 animals comprised the control group (GI), which was also divided into three groups of ten animals, which were also evaluated after 24 h, three days, and seven days (Table 1).

**Table 1:** Identification of studied samples with bismuth subgallate.

Experiment 24 h		Experiment three days		Experiment seven days	
Animal	Weight (g)	Animal	Weight (g)	Animal	Weight (g)
1	272.91	11	261.56	21	287.28
2	278.63	12	256.09	22	262.75
3	252.08	13	259.66	23	281.18
4	250.87	14	267.43	24	261.65
5	266.3	15	283.81	25	274.4
6	255.64	16	254.11	26	262.44
7	275	17	257.74	27	273.47
8	249.6	18	272.17	28	258.95
9	273.37	19	263.36	29	277.13
10	272.34	20	263.58	30	265.94

The control group was treated by washing the wound with 0.9% NaCl solution, as recommended by the literature. The fragment was removed with a margin of approximately 0.5 cm around the entire mucosal lesion, which went as deep as the buccal musculature of the rat. Immediately after the end of each surgery, the animals were euthanized by a lethal intraperitoneal dose of thiopental sodium (120 mg/kg). There was only one loss during the study, an animal from the control group, number 57 (Table 2), five days post-operatively.

**Table 2:** Identification of studied samples from the control group.

Control 24 h		Control three days		Control seven days	
Animal	Weight (g)	Animal	Weight (g)	Animal	Weight (g)
31	271.06	41	274.35	51	284.02
32	268.96	42	278.96	52	284.6
33	257.99	43	282.05	53	265.88
34	281.5	44	272.44	54	254.1
35	268.2	45	256.63	55	282.46
36	251.8	46	262.35	56	261.76
37	271.26	47	245.71	57	236.3
38	249.7	48	249.17	58	244.34
39	278.79	49	247.94	59	276.49
40	271.49	50	262.56	60	249.1

### Histological and immunohistochemical analysis

The study results were described as mean, median, minimum, maximum values, and standard deviations. The nonparametric Mann–Whitney test was used to compare the study and control groups regarding the percentage of collagen (mature and immature,) as well as the cell count score and the number of vessels. Comparisons made on the three days of assessment within each group were made using the nonparametric Kruskal–Wallis test. *p*-Values < 0.05 were considered statistically significant. Data were analyzed using the SPSS v. 20.0 software.

The section used for the histological analysis was placed on identified cardboard and secured with colored pins to schematize wound position in the rat. Subsequently, it was dipped in a container with 10% formaldehyde for 24 h. After that, it was embedded in paraffin blocks, and sectioned at 5-μm thick sections.

**Figure 3:**
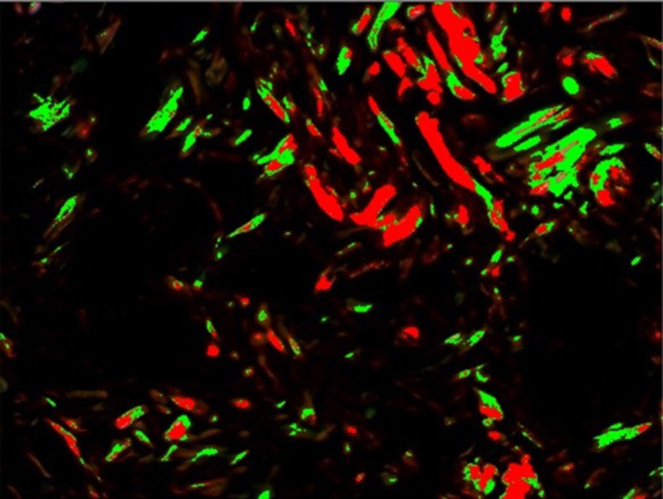
Mask used for the reading of Picrosirius staining.

**Figure 4:**
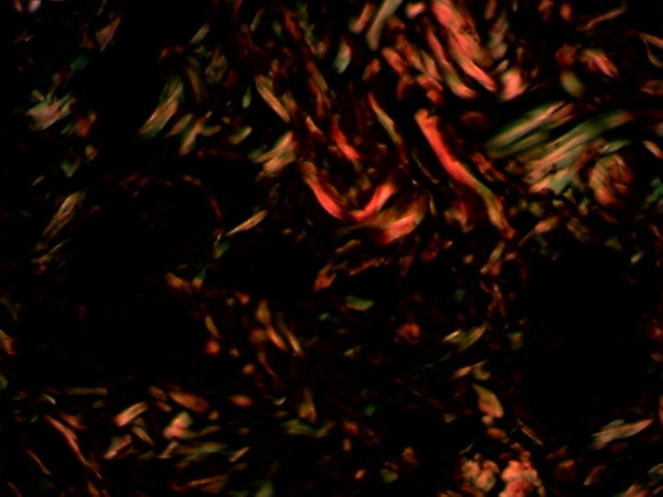
Slide showing Picrosirius staining of integra.

The histological sections were stained with hematoxylin-eosin and picrosirius. For each slide, the reading was performed in three fields for both stains, with 200× magnification, over the lesion area (Figure 3, Figure 4).

With hematoxylin-eosin stain, the inflammatory process was assessed, counting monomorphonuclear and polymorphonuclear cells and the number of vessels. The picrosirius stain assesses collagen through its specific birefringence, identifying the fiber orientation through a polarizing optical microscope. The thicker fibers, with greater birefringence, will appear colored in orange and red tones, identifying the type I collagen; the thinner fibers, with lower birefringence, will display a green color, identifying type III collagen.

**Figure 5:**
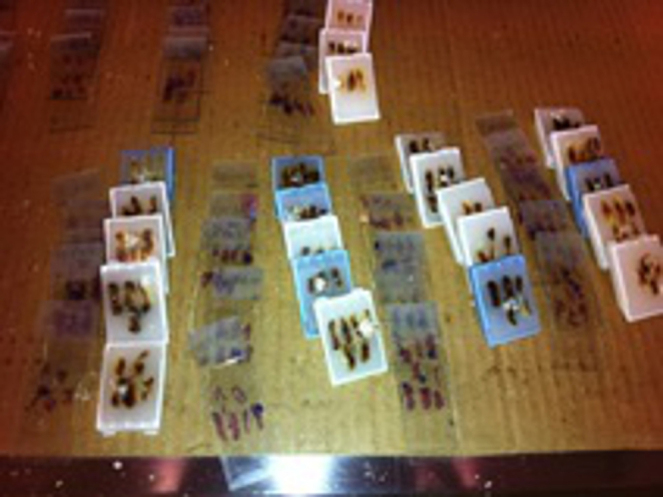
Finished slides and paraffin after punch was performed.

An Olympus BX50 microscope with a 3CCD Pro-S series capture cameras was used together with the Image-Pro Plus program, v. 4.5, by Cybernetics. The images were captured using a Dino-Lite camera and were analyzed using the application Image Plus 4.5 for Windows in Pentium III computer. After the reading, the areas with greater cellularity were defined, which were submitted to a punch, in order to prepare the tissue macroarray (TMA) for immunohistochemistry analysis (Fig. 5).

**Figure 6:**
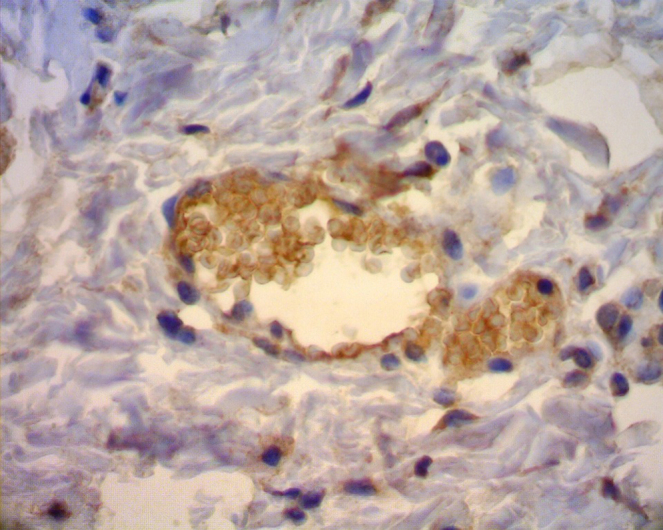
Mask used for the reading of CD34 marker.

**Figure 7:**
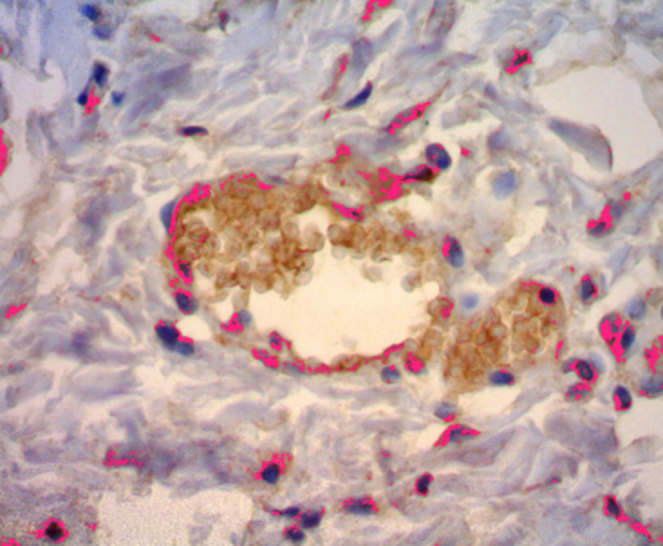
Slide showing CD34 marker of integra.

After TMA was performed, the material was sent for immunohistochemical processing. The CD34 marker was used, which allowed the angiogenesis process assessment by determining endothelial cell proliferation, as its antigen is a single-chain transmembrane glycoprotein associated with human hematopoietic progenitor cells (Fig. 6). The antigen-antibody reaction is defined by a brownish chemical reaction, allowing the quantification and qualification of the healing process (Fig. 7).

### Statistical analysis

The analysis was performed taking into account the study results, described as mean, median, minimum, maximum values, and standard deviations. The nonparametric Mann–Whitney test was used for the comparison of the study and control groups. Comparisons of the days of assessment within each group were performed using the nonparametric Kruskal–Wallis test.

## Results

Collagen is the main component of tissue extracellular matrix. It is structured into a dense and dynamic network, resulting from its constant deposition and resorption. Collagen degradation starts early and is very active during the inflammatory process; during this process, it is possible to evaluate the healing activity, mainly by investigating its immature form, collagen type III, which comprises approximately 40% of the granulation tissue.

### Mature collagen

Initially, on each assessment day (one, three, and seven), the null hypothesis that the mature collagen percentage was equal in both groups was tested *vs.* the alternative hypothesis of different mature collagen percentages (Table 3). Then, within each group, we tested the null hypothesis that the mature collagen percentage was the same on the three days of assessment *vs.* the alternative hypothesis that the percentages were not all the same. There was no difference regarding the mature collagen percentage in the experimental group on the assessed days (*p* = 0.180). The same occurred with the control group (*p* = 0.326).

**Table 3:** Comparison in relation to mature collagen. The table shows mean, median, minimum, maximum, and standard deviation. It also shows the *p*-values of statistical tests.

Day	Group	*n*	Mean	Median	Minimum	Maximum	SD	*p*-Value^a^
1	Study	10	82.3	86.5	59.6	95.5	12.2	
	Control	10	88.3	95.0	55.2	99.1	13.9	0.143
3	Study	10	86.2	87.5	63.4	99.2	10.0	
	Control	10	92.2	92.6	82.0	98.2	5.2	0.123
7	Study	9	91.7	93.7	82.2	97.9	5.2	
	Control	10	94.7	96.2	80.3	100.0	6.1	0.079

SD, standard deviation.

a Nonparametric Mann–Whitney test, *p* < 0.05.

### Immature collagen

Initially, on each assessment day (one, three, and seven), the null hypothesis that immature collagen percentage was equal in both groups was tested *vs.* the alternative hypothesis of different immature collagen percentages (Table 4). Then, within each group, the null hypothesis that immature collagen percentage was the same on the three days of assessment was tested *vs.* the alternative hypothesis that the percentages were not all the same. The results of immature collagen assessment showed no difference regarding its their percentage on the days assessed in the control (*p* = 0.326) and experimental groups (*p* = 0.180).

**Table 4:** Comparison in relation to immature collagen. The table shows mean, median, minimum, maximum, and standard deviation. *p*-Values of the statistical tests are also given.

Day	Group	*n*	Mean	Median	Minimum	Maximum	SD	*p*-Value^a^
1	Study	10	17.7	13.5	4.5	40.4	12.2	
	Control	10	11.7	5.0	0.9	44.8	13.9	0.143
3	Study	10	13.8	12.5	0.8	36.6	10.0	
	Control	10	7.8	7.4	1.8	18.0	5.2	0.123
7	Study	9	8.3	6.3	2.1	17.8	5.2	
	Control	10	5.3	3.8	0.0	19.7	6.1	0.079

SD, standard deviation.

a Nonparametric Mann–Whitney test, *p* < 0.05.

### Inflammatory process cell count

Initially, on each assessment day (one, three, and seven), the null hypothesis that the cell count score of the inflammatory process was equal in both groups was tested *vs.* the alternative hypothesis of different scores (Table 5).

**Table 5:** Comparison in relation to inflammatory cell growth. The table shows median, minimum, and maximum values. *p*-Values of statistical tests are also given.

Day	Group	*n*	Median	Minimum	Maximum	*p*-Value^a^
1	Study	10	0	−1	0	
	Control	10	0	−2	0	0.684
3	Study	10	0	−1	2	
	Control	10	0	−2	1	0.481
7	Study	9	1	0	3	
	Control	10	0	−1	1	0.043

a Nonparametric Mann–Whitney test, *p* < 0.05.

Then, within each group, the null hypothesis that the scores were the same on the three days of assessment was tested *vs*. the alternative hypothesis that the scores were not all the same. For the study group, the test results indicated rejection of the null hypothesis (*p* = 0.016). Considering that a significant difference was found between the days, they were compared two-by-two (Table 6).

**Table 6:** *p*-Value assessment. The table shows *p*-values of statistical tests.

Compared days	*p*-Value
Day one × day three	0.131
Day one × day seven	0.003
Day three × day seven	0.084

For the control group, the test results indicated the non-rejection of the null hypothesis (*p* = 0.289). Thus, in this group, there was no significant difference between the days of assessment in relation to the cell count score. The experimental group showed higher inflammatory reaction with increasing monomorphic proliferation (*p* = 0.016).

### Number of vessels

#### Hematoxylin eosin staining

Initially, on each day of assessment (one, three, and seven), the null hypothesis in which the number of vessels is the same in both groups was tested *vs*. the alternative hypothesis of different numbers of vessels (Table 7).

**Table 7:** Comparison in relation to the number of vessels. The table shows median, minimum, and maximum values. *p*-Values of statistical tests are also given.

Day	Group	*n*	Mean	Median	Minimum	Maximum	SD	*p*-Value^a^
1	Study	10	22.3	19.5	7.0	38.0	9.4	
	Control	10	20.2	19.0	2.0	50.0	13.4	0.529
3	Study	10	28.7	29.0	15.0	41.0	9.1	
	Control	10	43.2	39.5	12.0	70.0	18.6	0.052
7	Study	9	62.9	68.0	10.0	148.0	47.9	
	Control	10	53.8	48.5	11.0	120.0	31.2	0.968

SD, standard deviation.

a Nonparametric Mann–Whitney test, *p* < 0.05.

Then, within each group, the null hypothesis that the scores were the same on the three days of assessment was tested *vs*. the alternative hypothesis that the scores were not all the same. There was a greater proliferation of vessels in the control group (*p* = 0.006). The experimental group showed no significant vessel proliferation on the assessed days (*p* = 0.119; Table 8).

**Table 8:** *p*-Values of the statistical tests.

Compared days	*p*-Value
Day one × day three	0.007
Day one × day seven	0.001
Day three × day seven	0.480

#### CD34 marker

Initially, on each day of assessment (one, three, and seven), the null hypothesis that the CD34 result would be the same in both groups was tested *vs*. the alternative hypothesis of different results (Table 9). Then, within each group, the null hypothesis that the CD34 result was the same on the three days of assessment was tested *vs.* the alternative hypothesis that the results were not all the same.

**Table 9:** Comparison in relation to angiogenesis. The table shows median, minimum, and maximum values. *p*-Values of statistical tests are also given.

Day	Group	*n*	Mean	Median	Minimum	Maximum	SD	*p*-Value a
1	Study	10	6742	5949	1348	13,106	3626	
	Control	8	9371	7737	3341	21,206	5981	0.460
3	Study	9	5846	4937	2131	13,493	3735	
	Control	10	9406	8188	3814	15,493	3999	0.079
7	Study	9	4347	4419	619.8	7590	2281	
	Control	8	7575	6799	2437	15,750	3843	0.036

SD, standard deviation.

a Nonparametric Mann–Whitney test, *p* < 0.05.

There was greater proliferation in the control group on day seven (*p* = 0.036), whereas there was no significant proliferation of vessels in the experimental group.

## Discussion

Since bismuth subgallateis commonly used in medical practice, especially by otorhinolaryngologists and dermatologists, we designed this study to clarify the benefits of the salt in surgical procedures. Currently it is used commonly in tonsillectomies in several services, mainly in southern Brazil. The authors certainly have observed its benefits in their service; it provides clearer visualization of the bleeding points in oropharyngeal lesions, in addition to providing possible beneficial effects on healing and hemostasis.

The healing process involves several reactions occurring simultaneously; thus, it is not possible to separate the beginning and end of each stage. In theory, however, the reaction is divided into steps: coagulation, inflammation, proliferation, contraction, and remodeling.^1^ This process has the ultimate goal of tissue repair, but that requires all steps to occur satisfactorily.

Coagulation starts a few seconds after the creation of the wound. The clot and the extracellular matrix are formed; they contain chemical compounds (growth factor) and inflammatory mediators (cytokines) that contribute to the re-epithelialization and wound contraction processes.

The inflammatory process is characterized by hemostasis, cell migration, and provisional matrix deposition. Platelets are essential for the formation of the hemostatic plug that secretes multiple mediators, including growth factors released into the damaged area. Induced by thrombin, they also undergo platelet degranulation and release several growth factors, such as platelet-derived growth factor (PDGF), transforming growth factor β (TGF-β), epidermal growth factor (EGF), the transforming growth factor α (TGF-α), and the vascular endothelial growth factor (VEGF), in addition to adhesive glycoproteins, such as fibronectin and thrombospondin, which are important constituents of the provisional extracellular matrix.^5^, ^6^, ^8^

The macrophage is the most important cell in the healing process. In addition to a prominent role in phagocytosis of cellular debris, macrophages also secrete chemotactic factors that attract other inflammatory cells to the wound site and produce prostaglandins, which act as potent vasodilators affecting the permeability of microvessels. They also prepare the wound for the proliferative phase. The proliferation in healing scar formation promotes wound closure. This phase consists of re-epithelialization, fibroplasia, and angiogenesis.

It was decided in this study to focus on the process of angiogenesis, as it is crucial for healing and accounts for 60% of the repair tissue.^5^, ^6^ Stashak et al., cited by Souza et al.^9^ reported that angiogenesis shortens healing time by increasing the supply of nutrients and cell entry into the affected area, thus decreasing wound retraction time. Balbino et al.^10^ reported the migration of endothelial cells through the vessel wall where they then travel through the extracellular matrix toward the site of injury. Once in the outer region of the vessel, they undergo a differentiation process and acquire the capacity to form new capillary tubules. Migrating endothelial cells form a capillary sprout outside of the vessel, which joins the originating capillary, restoring blood flow.

The formation of the new vessel begins with tissue injury, with the activation of macrophages and the substances they produce. The proteases (plasmin and collagenases) digest the basement membrane and allow endothelial cells stimulated by angiogenic cytokines to form the new vascular bundle, which invades the wound. Angiogenesis ceases by apoptosis.^11^, ^12^

The specific mechanism responsible for contraction may be due to movement of myofibroblasts through the extracellular matrix. The latter have contractile actin and myosin fibers in their cytoplasm that originate from mesenchymal stem cells, circulating mononuclear cells or fibrocytes situated around the adventitia of local blood vessels, a process facilitated by TGF-β. Fibroblasts on the wound margins show a change in phenotype to myofibroblasts, with functional characteristics similar to those of smooth muscle. These cells are aligned around deposits of new extracellular matrix, establishing cell-to-cell connections and generating tensilestrength.^10^

Collagen modeling is a balance between the synthesis and degradation of collagen. This process is mediated by proteolytic enzymes (metalloproteinases), epidermal cells, endothelial cells, and fibroblasts.^11^ During remodeling, there are several stages of production, digestion, and orientation of the collagen fibers. The fibers are digested by collagenase, resynthesized, rearranged according to the array of adjacent connective tissue fibers, and connected laterally by covalent bonds. The amount of collagen increases during the first week, and by 21 days, there is a balance between production and degradation. Scar resistance is given by the amount of deposited collagen and the manner in which the fibers are arranged, with resistance being greater than it would be expected only by collagen deposition. This process begins around the third week after the injury and persists for months or years.^10^, ^13^

The objective for surgical recovery is for faster and more effective tissue recovery. The present study aimed to evaluate bismuth subgallateon wound healing, hoping to improve the response to trauma. Bismuth subgallate or bismuth oxygallate is a yellowish substance, which presents as an odorless powder that suffers discoloration in the presence of sunlight.^1^ It is a heavy metal salt, little adsorbed, and has strong astringent power.^2^

This compound has been used to contain hemorrhages in tonsillectomy, turbinectomy, and partial hepatectomy.^2^ Bismuth subgallate, a heavy metal, relatively insoluble, and poorly absorbed from the surgical bed, is inexpensive and easy to use, and is effective as a local hemostatic agent. Its mechanism of action is attributed to the activation of clotting factor 12, in addition to a presumed local astringent effect.^14^

Arroyo Júnior et al.^2^ also stated that bismuth subgallate acts in the activation of factor 12, which accelerates the intrinsic coagulation cascade pathway, contributing to the hemostatic characteristic of this compound. The authors discussed the possibility that bismuth subgallate can inhibit fibrinolysis and, thereby, increase fibrosis. This statement prompted us to design this study to demonstrate the contribution of bismuth subgallate to the healing process and, consequently, to increased formation of new vessels.

With respect to the inflammatory process as measured by the number of mononuclear and polymorphonuclear cells, the study demonstrated that the experimental group had reduced inflammatory process time, *i.e.*, the tissue was exposed to a faster inflammatory reaction, which did not result in the expected new cell formation.

The study demonstrated that bismuth subgallate promoted a significant difference in inflammatory pattern, and on the seventh day, the bismuth subgallate promoted a reduction in the inflammatory process, leading to a more marked chronic status than in the control group.

Results of the hematoxylin-eosin staining revealed that the bismuth subgallate did not promote an increase in vessels compared to the control group. But when comparing angiogenesis of the first with the seventh day in the control group, a significant increase in the number of vessels was observed. This association is expected during the normal physiological healing process. When angiogenesis was assessed by the immunohistochemical method with CD34 marker, a greater proliferation of vessels was observed in the control group, demonstrating that the subgallate inhibited neoangiogenesis in the experiment. The salt promoted a status of chronic inflammation, which may explain the inhibition of angiogenesis compared to the control group.

Collagen activity was another factor evaluated in the present study. It was affirmed that collagen is the most abundant connective tissue protein in the healing phase.^15^ This protein primarily comprises the extracellular matrix and is part of the remodeling phase of healing.

Type I collagen is the most common collagen type; it is synthesized by fibroblasts and is most prevalent in bones and tendons. Type III is most commonly found in soft tissues, such as blood vessels, dermis, and fascia. The healthy dermis contains approximately 80% of type I collagen and 20% type III collagen. Granulation tissue expresses 30–40% of type III collagen, immaturecollagen.^15^

There was no difference in collagen production, as the control and experimental groups showed the same efficiency.

## Conclusion

A possible negative effect of bismuth subgallate on the healing process in the mucosal wounds of the assessed animals was observed. The salt resulted in less tissue exposure to the inflammatory process, leading to an early chronic status with some stabilization in the number of vessels, thereby even delaying the physiological healing process and impairing angiogenesis.

The bismuth subgallate stimulus to fibroplasia was indifferent, as it did not interfere in collagen formation, either in its immature or mature form.

But when considering the entirety of the healing process, we demonstrated that the subgallate does not improve the quality of healing, and the heavy metal also interferes by delaying the rate of new vessel formation and optimal healing of the surgical wound.

## Conflicts of interest

The authors declare no conflicts of interest.
